# The Equilibria of Sphingolipid-Cholesterol and Sphingolipid–Sphingolipid in Monolayers at the Air–Water Interface

**DOI:** 10.1007/s00232-012-9496-5

**Published:** 2012-08-17

**Authors:** Aneta Dorota Petelska, Zbigniew Artur Figaszewski

**Affiliations:** 1 Institute of Chemistry, University of Bialystok, Al. J. Pilsudskiego 11/4, 15-443 Białystok, Poland; 2 Laboratory of Electrochemical Power Sources, Faculty of Chemistry, University of Warsaw, Pasteur St. 1, 02-093 Warsaw, Poland

**Keywords:** Sphingomyelin, Ceramide, Cholesterol, Complex formation, Equilibria, Monolayer, Langmuir trough

## Abstract

Monolayers of sphingomyelin (SM), ceramide (Cer) and cholesterol (Ch) and binary mixtures SM–Ch, SM–Cer and Cer–Ch were investigated at the air–water interface. SM, Cer and Ch were used in the experiment. The surface tension values of pure and mixed monolayers were used to calculate π-A isotherms. Surface tension measurements were carried out at 22 °C using a Teflon trough and a Nima 9000 tensiometer. Interactions between sphingolipid and Ch as well as sphingolipid and another sphingolipid result in significant deviations from the additivity rule. An equilibrium theory to describe the behavior of monolayer components at the air–water interface was developed in order to obtain the stability constants and Gibbs free energy values of SM–Ch, SM–Cer and Cer–Ch complexes. We considered the equilibrium between the individual components and the complex and established that sphingolipid and Ch as well as sphingolipid and another sphingolipid formed highly stable 1:1 complexes.

## Introduction

Cholesterol (Ch), ceramide (Cer) and sphingomyelin (SM) are important constituents of cellular plasma membranes. These molecules are chemically as well as functionally different, and still they appear to colocalize in the same membrane compartment and even to be attracted to each other. Cers are relatively minor polar components of cell membranes, varying between 1 and 10 % in proportion to glycerophospholipids (Hannun 1996). Despite being minor constituents, they are known to be mediators of vital cellular processes such as apoptosis, aging, differentiation and cell growth (Hancock 2006; Manes and Viola 2006; Morales et al. 2007).

Researchers for many years have been trying to define the physical properties of mixtures of Ch and sphingolipids in monolayer membranes. This quasi-2D system is of intrinsic interest to physical chemists. Studies on the monolayer lipid membranes also help us to understand certain properties of the membranes of animal cells (Slotte 1999; Bernholz 2004; Epand and Epand 2004; Halling and Slotte 2004; Guan et al. 2009).

Sphingolipid–Ch mixtures have been extensively investigated as models of the lipid bilayer regions of animal cell membranes (Radhakrishnan et al. 2001; Ohvo-Rekila et al. 2002). Studies of these mixtures in lipid monolayers have been used due to their simplicity and the ease with which the molecular density can be varied through changes of applied pressure. At lower pressures many mixtures of phospholipids and Ch form two coexisting liquid phases (Subramaniam and McConnell 1987; Slotte 1999; McConnell and Radhakrishnan 2003).

There is clear biophysical evidence that sterols and sphingolipids can segregate from other lipids in simple artificial membrane systems to form liquid-ordered domains (Ahmed et al. 1997). Sterol partitioning experiments between membranes in vitro also suggest that they have affinity for membranes with a high content of sphingolipids (Wattenberg and Silbert 1983). Sterols have been shown to have a condensing effect on artificial membranes (Radhakrishnan and McConnell 2005), and sterol–SM condensed complexes have been characterized (Radhakrishnan et al. 2001). Some evidence in yeast suggests a genetic interaction between mutants in sterol and sphingolipid biosynthesis (Baudry et al. 2008; Eisenkolb et al. 2002; Jin et al. 2008); however, little, if any, convincing evidence exists to show that these two lipid species function together in complex biological membranes. Therefore, the basic question of whether sterols and sphingolipids interact functionally and preferentially in biological systems remains unsolved and is a major focus of this study.

Nevertheless, various lines of evidence suggest a possible link between these two lipid classes. Sterols and sphingolipids are concomitantly affected in certain diseases. In Niemann-Pick disease, although the primary defect is not yet completely certain, defects in sphingolipid and Ch trafficking appear to be interdependent (Puri et al. 1999; Pagano et al. 2000; Vance 2006). One of the proposed functions of amyloid-beta and presenilin is control of SM and Ch amounts in the brain (Grimm et al. 2005), which could affect the ontology of Alzheimer disease. Sphingolipid depletion has also been shown to influence the SREBP pathway, controlling Ch and lipid biosynthesis (Scheek et al. 1997).

Ceramide and SM differ in their affinity for interacting with other lipids that make up the membrane bilayer matrix, especially Ch. SM interacts with Ch to create a liquid-ordered structure, whereas Cer shows a relatively weak affinity toward Ch. Moreover, Cer exhibits a tendency to segregate into domains highly enriched in Cer (Veiga et al. 1999; Kolesnick et al. 2000; Hartel et al. 2005).

Cholesterol tends to destabilize the Cer-rich domains formed in phosphatidylcholine, while SM, by formation of stable complexes with Cer, tends to stabilize these domains. The stability of SM–Cer complexes is evident from the persistence of highly ordered structures probed by ESR spectroscopy and the appearance of a sharp, wide-angle X-ray reflection at temperatures higher than the gel–fluid transition of Cer alone in egg phosphatidylcholine bilayers. The competition between Cer and Ch for interaction with SM is discussed in terms of control of lipid-mediated signaling pathways by SMase and phospholipase A_2_ (Staneva et al. 2008).

The aim of the present work was to examine the possible effect of Ch or sphingolipid components on the sphingolipid monolayer and the molecular interaction between sphingolipid and Ch or another sphingolipid by analyzing physicochemical properties for binary mixed monolayers (sphingolipid–Ch, sphingolipid–another sphingolipid), treated as the simplest model of a half of the biological membrane. This work continues the systematic study of physicochemical properties of mono- and bilayer membranes realized by Figaszewski and co-workers (Naumowicz et al. 2006; Petelska and Figaszewski 2003, 2009, 2011; Petelska et al. 2006). We present evidence for the formation of 1:1 SM–Ch, SM–Cer and Cer–Ch complexes at the air–water interface and calculate their stability constants and Gibbs free energy values. The knowledge of stability constants and Gibbs free energy values of sphingolipid–Ch or sphingolipid–another sphingolipid systems allows us to understand the processes that take place both in the monolayer itself and on its surface. The results can be used in quantitative descriptions of the physical and chemical properties of biological membranes.

## Theory

During formation of a mixed two-component monolayer on a free electrolyte surface, the individual components (denoted A and B) can form complexes. The equilibrium of such a system is described by the complexation reaction. Two substances can form complexes of varying stoichiometry. However, due to the fact that the first stability constant in complexes is usually the largest (Inczedy 1976), we assumed that 1:1 complexes were predominant.

Let us assume that a 1:1 complex is formed in a mixed monolayer at the air–water interface. The reaction

$$ {\text{A}} + {\text{B}} \Leftrightarrow {\text{A}}{\text{B}} $$

may be described by the system of equations (Petelska and Figaszewski 2009, 2011):

1 $$ a_{\text{A}} S_{\text{A}} + a_{\text{B}} S_{\text{B}} + a_{\text{AB}} S_{\text{AB}} = 1 $$

2 $$ a_{\text{A}} + a_{\text{AB}} = c_{\text{A}} $$

3 $$ a_{\text{B}} + a_{\text{AB}} = c_{\text{B}} $$

4 $$ K_{\text{AB}} = \frac{{a_{\text{AB}} }}{{a_{\text{A}} \cdot a_{\text{B}} }} $$

5 $$ x_{\text{B}} = \frac{{c_{\text{B}} }}{{c_{\text{A}} + c_{\text{B}} }} $$

where $a_{\text{A}}$, $a_{\text{B}}$ and $a_{\text{AB}}$ (mol m^−2^) are the surface concentrations of components A and B and complex AB; $c_{\text{A}}$ and $c_{\text{B}}$ (mol m^−2^) are the total surface concentrations of components A and B and complex AB; $S_{\text{A}}$, $S_{\text{B}}$ and $S_{\text{AB}}$ (m^2^ mol^−1^) are the surface areas occupied by 1 mol of components A and B and complex AB; $K_{\text{AB}}$ (m^2^ mol^−1^) is the stability constant of complex AB; and $x_{\text{A}}$ and $x_{\text{B}}$ are the mole fractions of components A and B.

The above system of Eqs. (1–5) contains unknown quantities, $a_{\text{A}}$, $a_{\text{B}}$, $a_{\text{AB}}$, $S_{\text{AB}}$ and $K_{\text{AB}}$, as well as quantities that are known or easy to calculate, $S_{\text{A}}$, $S_{\text{B}}$, $x_{\text{B}}$, $c_{\text{A}}$ and $c_{\text{B}}$.

Attempts to solve this system of equations resulted in complicated expressions, so Eqs. (1–5) were differentiated with respect to $x_{\text{B}}$ and approximated to low or high argument values. At $x_{\text{B}} \to 0$ (a monolayer formed from pure component A, $x_{\text{A}} \to 1$), the system of equations is simplified:

6 $$ a_{\text{A}}^{\prime } S_{\text{A}} + a_{\text{B}}^{\prime } S_{\text{B}} + a_{\text{AB}}^{\prime } S_{\text{AB}} = 0 $$

7 $$ a_{\text{A}}^{\prime } + a_{\text{AB}}^{\prime } = c_{{{\text{A}}(x_{\text{B}} = 0)}}^{\prime } $$

8 $$ a_{\text{B}}^{\prime } + a_{\text{AB}}^{\prime } = c_{{{\text{B}}(x_{\text{B}} = 0)}}^{\prime } $$

9 $$ a_{\text{AB}}^{\prime } = K_{\text{AB}} \frac{1}{{S_{\text{A}} }}a_{\text{B}}^{\prime } $$

At $x_{\text{B}} \to 1$ (a monolayer formed from pure component B, $x_{\text{A}} \to 0$), the system of equations after differentiation with respect to $x_{\text{B}}$ is simplified:

10 $$ a_{\text{A}}^{\prime } S_{\text{A}} + a_{\text{B}}^{\prime } S_{\text{B}} + a_{\text{AB}}^{\prime } S_{\text{AB}} = 0 $$

11 $$ a_{\text{A}}^{\prime } + a_{\text{AB}}^{\prime } = c_{{{\text{A}}(x_{\text{B}} = 1)}}^{\prime } $$

12 $$ a_{\text{B}}^{\prime } + a_{\text{AB}}^{\prime } = c_{{{\text{B}}(x_{\text{B}} = 1)}}^{\prime } $$

13 $$ a_{\text{AB}}^{\prime } = K_{\text{AB}} \frac{1}{{S_{\text{B}} }}a_{\text{A}}^{\prime } $$

In the above equations, $a_{\text{A}}^{\prime }$, $a_{\text{B}}^{\prime }$ and $a_{\text{AB}}^{\prime }$ are the derivatives of $a_{\text{A}}$, $a_{\text{B}}$ and $a_{\text{AB}}$ with respect to $x_{\text{B}}$.

The quantities $a_{\text{A}}^{\prime }$, $a_{\text{B}}^{\prime }$ and $a_{\text{AB}}^{\prime }$ can be eliminated from the system of equations if the values of $S_{\text{A}}$ and $S_{\text{B}}$ are known. Suitable transformations lead to expressions for two quantities of interest: the stability constant of the complex, $K_{\text{AB}}$, and the surface area occupied by one molecule of the complex, $S_{\text{AB}}$:

14 $$ K_{\text{AB}} = \frac{{S_{\text{B}}^{3} c_{{{\text{B}}(x_{\text{B}} = 1)}}^{\prime } - 2S_{\text{A}} S_{\text{B}} - S_{\text{A}}^{3} c_{{{\text{A}}(x_{\text{B}} = 0)}}^{\prime } }}{{S_{\text{B}} - S_{\text{A}} + S_{\text{A}}^{2} c_{{{\text{A}}(x_{\text{B}} = 0)}}^{\prime } + S_{\text{B}}^{2} c_{{{\text{B}}(x_{\text{B}} = 1)}}^{\prime } }} $$

15 $$ S_{\text{AB}} = \frac{{\left( {S_{\text{A}} S_{\text{B}} + c_{{{\text{A}}(x_{\text{B}} = 0)}}^{\prime } c_{{{\text{B}}(x_{\text{B}} = 1)}}^{\prime } S_{\text{A}}^{2} S_{\text{B}}^{2} } \right)\left( {S_{\text{A}} + S_{\text{B}} } \right)}}{{S_{\text{A}}^{3} c_{{{\text{A}}(x_{\text{B}} = 0)}}^{\prime } + S_{\text{B}}^{3} c_{{{\text{B}}(x_{\text{B}} = 1)}}^{\prime } }} $$

The slopes of tangent lines at the points $x_{\text{B}} = 0$ (pure component A) and $x_{\text{B}} = 1$ (pure component B) may be calculated from the following equations:

16 $$ c_{{{\text{A}}(x_{\text{B}} = 0)}}^{\prime } = \frac{{K_{\text{AB}} \left( {S_{\text{A}} - S_{\text{AB}} } \right) - S_{\text{A}} S_{\text{B}} }}{{S_{\text{A}}^{2} \left( {S_{\text{A}} + K_{\text{AB}} } \right)}} $$

17 $$ c_{{{\text{B}}(x_{\text{B}} = 1)}}^{\prime } = \frac{{ - K_{\text{AB}} \left( {S_{\text{B}} - S_{\text{AB}} } \right) - S_{\text{A}} S_{\text{B}} }}{{S_{\text{B}}^{2} \left( {K_{\text{AB}} - S_{\text{B}} } \right)}} $$

Equations 16 and 17 may be used for verification of the slopes obtained either from theory or by experiment. Agreement between the slopes indicates that the method of calculating $K_{\text{AB}}$ and $S_{\text{AB}}$ is correct.

## Materials and Methods

### Materials

SM from chicken egg yolk ≥98 % (TLC), Cer from bovine brain ≥98 % (TLC) and Ch from hog liver ≥97 % (GC) were purchased from Fluka (Buchs, Switzerland) and used as received. Cer was prepared from cerebrosides by a modification of the procedure presented in Carter et al. (1961). The molecular weights of SM, Cer and Ch were approximately 731.09, 563.95 and 386.67 g mol^−1^, respectively.

The 1-chloropropane solvent (>98 % pure) was supplied by Aldrich (Milwaukee, WI). Solutions were prepared by dissolving appropriate amounts of each material in 1-chloropropane at a concentration of 1 mg cm^−3^ and stored at 4 °C. The water used in the experiments was prepared by triple distillation (the second distillation was performed over KMnO_4_ and KOH to remove organic impurities).

### Methods

The homemade, computer-controlled apparatus used for surface tension measurements was presented previously (Petelska and Figaszewski 2009).

Surface tension measurements were carried out at the water–air interface at 22 °C and expressed as surface pressure–area per molecule (π-A) isotherms. For all experiments, the trough was filled with triple-distilled water as the subphase. Monolayers were prepared by spreading a defined volume of a lipid solution in 1-chloropropane on the aqueous subphase using a Hamilton (Reno, NV) microsyringe. 10 min were allowed for solvent evaporation and monolayer equilibration before an experiment was begun. The monolayer was continuously compressed to obtain the π-A isotherms using the glass barrier. The Nima ST9002 computer program was used to calculate the surface pressure of the monolayer π as a function of surface area per molecule A: π = γ − γ_*0*_ = *f*(*A*), where γ is the surface tension of the lipid-covered surface and γ_*0*_ is the surface tension of the bare air–water interface.

Before each trial the Teflon trough (trough size 648 cm^2^) was washed and rinsed with purified water. The subphase surface was cleaned just prior to each measurement by suction with a vacuum pump until the surface tension was constant and equal to the surface tension value of pure water at 22 °C (approximately 72 mN m^−1^). All glassware in contact with the samples was cleaned with chromic acid and repeatedly rinsed with purified water before use.

The system was enclosed in an acrylic box to minimize water evaporation, to ensure high humidity and to avoid contamination.

All of the reported values are highly reproducible and represent the average of at least five experiments. The SD for surface area measurements was <1 %.

## Results and Discussion

We present evidence for the formation of 1:1 SM–Ch, SM–Cer and Cer–Ch complexes at the air–water interface. Using equations from the Theory section, the stability constants and Gibbs free energy of the SM–Ch, SM–Cer and Cer–Ch complexes were calculated. This is the first report of stability constants and Gibbs free energies for SM–Ch, SM–Cer and Cer–Ch complexes in monolayer.

**Fig. 1:**
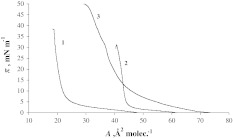
Isotherms of Cer (*1*), Ch (*2*) and SM (*3*)

Figure 1 presents π-A isotherms of Cer (1), Ch (2) and SM (3). The slopes of Cer and Ch isotherms are very high, indicating a perpendicular orientation of the molecules at the interface with the hydrophilic group directed at the aqueous subphase. The Ch isotherm is in satisfactory agreement with that previously reported (Brzozowska and Figaszewski 2002; Walker et al. 2008; Petelska and Figaszewski 2009). The surface areas for Cer and Ch molecules (22 ± 0.2 and 46 ± 0.5 Å^2^ molecule^−1^) were obtained experimentally by extrapolating isotherms to π = 0. This is in agreement with the previously reported values (Kamel et al. 1971; Brzozowska and Figaszewski 2002; Walker et al. 2008).

The π-A isotherm of SM (denoted by 3 in Fig. 1) is shaped differently. SM monolayer is an example of a liquid-expanded membrane, with the hydrophilic headgroup located in the aqueous subphase and the hydrophobic fatty acid tails oriented toward the air. The SM isotherm is in satisfactory agreement with that previously reported (Prenner et al. 2007). The surface pressure–area (π-A) isotherm of pure SM indicated a phase transition from the liquid-expanded to a liquid-condensed state at around 16 mN m^−1^, in agreement with published data (Smaby et al. 1994; Prenner et al. 2007). Additionally, a second phase transition from the liquid-condensed to a solid phase, characterized by a far steeper slope, can be deduced from the change in the slope of the isotherm around 35 mN m^−1^. The appearance of a solid phase is supported by the observation that the Wilhelmy plate started to incline at pressures close to the collapse of the monolayer around 50 mN m^−1^. Furthermore, if the compression was stopped in this surface pressure range, the Wilhelmy plate remained in the inclined position (Smaby et al. 1994; Prenner et al. 2007).

The surface area for the SM molecule is 45 ± 0.5 Å^2^ molecule^−1^. The literature values range between 40 and 55 Å^2^ molecule^−1^ for SM (Kamel et al. 1971; Shaikh et al. 2001; Chiu et al. 2003).

### SM–Ch Complex

**Fig. 2:**
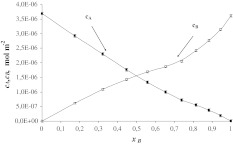
Dependence of total surface concentration of SM (*c*_A_) and Ch (*c*_B_) on the mole fraction of Ch (experimental values are indicated by *points* and theoretical values, by the *curve*)

The total surface concentrations of SM (*c*_A_) and Ch (*c*_B_) versus mole fraction of Ch are depicted in Fig. 2. The nearly linear shape of the *c*_B_ = *f*(*x*_B_) function confirms the condensed character of the membrane (Birdi 1989). The condensation effect of Ch describes the decrease in surface area per phospholipid molecule in the monolayer in the presence of Ch (Yeagle 1985). It is remarkable that the function *c*_B_ = *f*(*x*_B_) is almost linear for *x*_B_ > 0.5.

Such interactions in the investigated sphingolipid–Ch system can be explained in terms of complexes (Radhakrishnan et al. 2001; Ohvo-Rekila et al. 2002; Radhakrishnan and McConnell 2005). It was demonstrated by Radhakrishnan et al. (2001) that the complex was formed in the SM–Ch system. The 1:1 SM–Ch complex has been assumed to exist in monolayers composed of SM and Ch (Eqs. 1–3). It is characterized by the stability constant *K*_AB_ (Eq. 4).

The area per SM–Ch complex, *S*_AB_ = 5.36 × 10^5^ m^2^ mol^−1^ (89 ± 0.9 Å^2^ molecule^−1^), and the stability constant, *K*_AB_ = 4.02 × 10^5^ m^2^ mol^−1^, were calculated by inserting the experimental data into Eqs. 14 and 15. It should be emphasized that the stability constant is higher for complexes in bilayers (*K* = 1.61 × 10^8^ m^2^ mol^−1^) (Petelska et al. 2009). A monolayer is a 2D system forming a plane at the air–water interface, while a bilayer possesses a 3D and is additionally stabilized by hydrophobic interactions between the hydrocarbon chains.

The *S*_AB_ value obtained this way is higher than the area of an SM molecule (*S*_A_ = 45 ± 0.5 Å^2^ molecule^−1^) but slightly lower than the sum of the areas of SM and Ch (*S*_A_ + *S*_B_ = 91 ± 1.0 Å^2^).

Using the values calculated for *S*_AB_ and *K*_AB_ in Eqs. 16 and 17, theoretical $c_{\text{A}}^{\prime }$ and $c_{\text{B}}^{\prime }$ values were calculated and compared with the slopes of lines tangent to the experimental data at points *x*_B_ = 0 and *x*_B_ = 1.

### SM–Cer and Cer–Ch Complexes

**Fig. 3:**
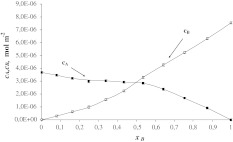
Dependence of total surface concentration of SM (*c*_A_) and Cer (*c*_B_) on the mole fraction of Cer (experimental values are indicated by *points* and theoretical values, by the *curve*)

**Fig. 4:**
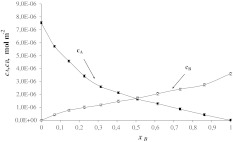
Dependence of total surface concentration of Cer (*c*_A_) and Ch (*c*_B_) on the mole fraction of Ch (experimental values are indicated by *points* and theoretical values, by the *curve*)

Figures 3 and 4 present the total surface concentrations of SM (*c*_A_) and Cer (*c*_B_) versus mole fraction of Cer (*c*_A_) and Ch (*c*_B_) as a function of the Ch mole fraction. In monolayers composed of SM and Cer or Cer and Ch (Eqs. 1–3) it has been assumed that 1:1 SM–Cer and Cer–Ch complexes exist. These complexes are characterized by the stability constants *K*_AB_ (Eq. 4), which were *K*_AB_ = 6.75 × 10^4^ m^2^ mol^−1^ (SM–Cer) and 2.61 × 10^5^ m^2^ mol^−1^ (Cer–Ch). The stability constants presented above were calculated by inserting the experimental data into Eq. 14. It should be emphasized that the stability constants are higher for these complexes in bilayers (*K* = 1.47 × 10^7^ m^2^ mol^−1^ for SM–Cer complex and *K* = 8.30 × 10^7^ m^2^ mol^−1^ for Cer–Ch complex) (Petelska et al. 2009).

The areas per SM–Cer and Cer–Ch complexes, *S*_AB_ = 5.12 × 10^5^ m^2^ mol^−1^ (85 ± 0.8 Å^2^ molecule^−1^) and *S*_AB_ = 3.98 × 10^5^ m^2^ mol^−1^ (66 ± 0.7 Å^2^ molecule^−1^), respectively, were calculated by inserting the experimental data into Eq. 15. The *S*_AB_ values for SM–Cer obtained in this way are higher than the area of an SM molecule (*S*_A_ = 46 ± 0.5 Å^2^ molecule^−1^) but slightly lower than the sum of the areas of SM and Cer (*S*_A_ + *S*_B_ = 67 ± 0.7 Å^2^), but the *S*_AB_ value for Cer–Ch is equal to the sum of the areas of Cer and Ch (*S*_A_ + *S*_B_ = 68 ± 0.7 Å^2^).

Using the values calculated for *S*_AB_ and *K*_AB_ in Eqs. 16 and 17, theoretical $c_{\text{A}}^{\prime }$ and $c_{\text{B}}^{\prime }$ values were calculated and compared with the slopes of lines tangent to the experimental data at points *x*_B_ = 0 and *x*_B_ = 1.

Our results show that addition of Ch to the membrane constructed from sphingolipids resulted in increased stability and reproducibility of the membranes. Ch condenses some membrane components (condensing effect), making the membrane structures more rigid. It also improves the packing of membrane lipids as they occupy more places in the hydrophobic layer of the membrane and fewer places in the polar groups, unlike the sphingolipid molecules.

In Figs. 2, 3, and 4, the experimental points are compared with the values calculated using Eqs. 1–3 (depicted as lines). The theoretical values obtained are presented in Figs. 2, 3, and 4 and are marked by lines; points on the same figure show the experimental values. It can be seen that the agreement between experimental and theoretical points is very good, which verifies the assumption of the formation of 1:1 complexes in the mixed SM–Ch, SM–Cer and Cer–Ch monolayers. The lack of variation between theoretical and experimental points indicates that the theoretical model (presented under Theory, above) is sufficient to describe the interaction in sphingolipid–Ch and sphingolipid–another sphingolipid systems. The agreement between the experimental results and the model predictions for these systems justifies the statement that other complexes do not represent a significant component of these systems.

Table 1 lists several physicochemical parameters for monolayers containing SM–Ch, SM–Cer and Cer–Ch complexes.

**Table 1:** Selected physicochemical parameters for three complexes: SM–Ch, SM–Cer and Cer–Ch

Examined system	Surface area occupied by one molecule of complex (Ǻ^2^ molecule^−1^)	Stability constant of examined complex (m^2^ mol^−1^)	Complex formation energy (Gibbs free energy) (kJ mol^−1^)
SM–Ch	89 ± 0.9	4.02 × 10^5^	−31.72 ± 1.35
SM–Cer	85 ± 0.8	6.75 × 10^4^	−27.33 ± 1.24
Cer–Ch	66 ± 0.7	2.61 × 10^5^	-30.66 ± 1.32

## Conclusions

Analysis of the results presented in Table 1 leads to the following conclusions:

1. The stability constant of the SM–Ch complex is 4.02 × 10^5^ m^2^ mol^−1^, whereas the stability constant of the SM–Cer and Cer–Ch complexes are 6.75 × 10^4^ m^2^ mol^−1^ and 2.61 × 10^5^ m^2^ mol^−1^, respectively. These values are relatively high, providing additional support for the prevalence of 1:1 complexes in mixed monolayers.
2. The experimentally obtained value for the area occupied by the SM–Ch complex is 89 ± 0.9 Å^2^ molecule^−1^, the area occupied by SM–Cer complex is 85 ± 0.8 Å^2^ molecule^−1^ and the area occupied by Cer–Ch complex is 66 ± 0.7 Å^2^ molecule^−1^.
3. The complex formation energy (Gibbs free energy) values for the SM–Ch, SM–Cer and Cer–Ch complexes are −31.72 ± 1.35, −27.33 ± 1.24 and −30.66 ± 1.32 kJ^ ^mol^−1^, respectively.
4. The excellent agreement between the experimental and theoretical points validates the assumption of 1:1 complex formation in the sphingolipid monolayer. The homogeneity of the measurement results indicates that complexes of stoichiometries other than 1:1 do not play a significant role in these systems.
